# Recurrent Liver Abscess in a Non-toxic Patient

**DOI:** 10.7759/cureus.33929

**Published:** 2023-01-18

**Authors:** Ayham Khrais, Ahmad S Ali, Catherine Choi, Siddharth Verma

**Affiliations:** 1 Department of Medicine, Rutgers University New Jersey Medical School, Newark, USA; 2 Department of Neurology, Rutgers University New Jersey Medical School, Newark, USA; 3 Department of Gastroenterology and Hepatology, Rutgers University New Jersey Medical School, Newark, USA; 4 Department of Gastroenterology and Hepatology, East Orange Veteran's Affairs Medical Center, East Orange, USA

**Keywords:** hepatic lesion, asymptomatic disease, e. coli, bacterial liver abscess, pyogenic abscess

## Abstract

Liver abscesses range in presentation from asymptomatic infection to sepsis. Recurrence is rare. We describe a case of an asymptomatic liver abscess that recurred 10 years after a previous abscess. The patient presented with flu-like symptoms and dark urine. Laboratory evaluation showed an elevation of aminotransferases and bilirubin. Triple-phase CT showed a 2.8 cm mass in the right liver lobe with linear enhancement. The abscess was aspirated, with cultures growing *Escherichia coli*. The patient was started on ceftriaxone and metronidazole and then discharged with outpatient follow-up. We describe an unusual case of asymptomatic pyogenic liver abscess growing *E. coli*, with the same location and causative organism as an abscess that occurred 10 years prior.

## Introduction

Liver abscesses are the most common type of visceral abscess with a yearly incidence of 2.3 cases per 100,000 individuals [1]. Risk increases with age, male sex at birth, diabetes, malignancy, and history of liver transplant [2]. Pyogenic liver abscesses (PLAs) can arise from two main etiological pathways: direct inoculation of bacteria via an adjacent biliary tract infection, such as cholangitis, bile tract obstruction, or cholecystitis can occur, resulting in the seeding of intrahepatic vessels and parenchyma, with eventual abscess formation within the liver itself. An alternate mechanism involves the development of portal vein septicemia, either by peritonitis, leakage of bowel contents or via instrumentation-induced injury to intra-abdominal contents [3]. Finally, abscesses can result from bacteremia-induced seeding of the liver via the hepatic artery [4]. Most cases occur via a direct spread of microorganisms from within the biliary tree as a result of biliary tract pathology. The most commonly isolated organisms from PLAs are *Escherichia coli*, *Klebsiella pneumoniae*, *Streptococcus,* and *Enterococcus* species, with many abscesses being polymicrobial in composition as well [3,4].

The most common initial presenting symptoms of PLAs are subjective fever, chills, right upper quadrant (RUQ) abdominal pain, nausea, vomiting, weight loss, and diarrhea [5]. Associated laboratory abnormalities include elevation of liver function tests (LFTs), including aspartate aminotransferase (AST), alanine aminotransferase (ALT) & alkaline phosphatase (ALP), and leukocytosis with neutrophilic predominance [5]. Management involves intravenous antibiotics with or without drainage, in a case-dependent manner. We describe an unusual case of an asymptomatic spontaneous PLA recurring 10 years after a prior abscess, both having risen in the same location within the liver.

This article was previously presented as a poster at the American College of Gastroenterology Conference on October 24, 2022. Abstracts accepted at the conference were published in a special supplement of the October 2022 issue of The American Journal of Gastroenterology.

## Case presentation

A 73-year-old-man with a history of horseshoe kidney complicated by chronic kidney disease (CKD) stage 3, type 2 diabetes mellitus, hypertension, coronary artery disease status post coronary artery bypass grafting and percutaneous intervention, and abdominal aortic aneurysm status post endovascular aneurysm repair four years prior presented with dark urine for the past 2 weeks. The patient initially attributed these symptoms to a COVID-19 booster and influenza vaccinations that he received 14 days ago, where he developed flu-like symptoms with a maximum temperature of 101.8°F that took five days to resolve. Afterward, he noticed his urine becoming dark amber. He denied dysuria or hematuria. Over the next several days, he experienced generalized weakness and decreased oral intake. He called his primary care provider and was told to increase oral fluid intake and complete a laboratory evaluation. Blood work showed elevated liver function tests (LFTs), and he was told to present to the emergency department. Of note, the patient was not up to date with his colorectal cancer screening, and no records regarding his last colonoscopy were available.

On arrival, the patient was afebrile with a heart rate of 103 beats per minute, and a respiratory rate of 22 breaths per minute. The remaining vital signs were unremarkable. Physical examination was notable for mild yellowing of the bilateral upper extremities but no overt signs of jaundice. Laboratory evaluation (Table 1) was remarkable for a hemoglobin of 11.7 (reference range [RR]: 14-17.5 g/dL), sodium of 133 (RR: 135-145 mEq/L), creatinine of 1.4 (baseline 1.3; RR: 0.7-1.3 mg/dL), aspartate aminotransferase (AST) of 117 (RR: 8-33 U/L), alanine aminotransferase (ALT) of 212 (RR: 7-55 U/L), alkaline phosphatase (ALP) of 825 (RR: 44-147 U/L), total bilirubin of 4.1 (RR: 0.1-1.2 mg/dL), and direct bilirubin of 2.1 (RR: <0.3 mg/dL).

**Table 1:** Serum laboratory evaluation.

Laboratory Value		Reference Range
Hemoglobin	11.7	14-17.5 g/dL
Sodium	133	135-145 mEq/L
Creatinine	1.4	0.7-1.3 mg/dL
Aspartate Aminotransferase	117	8-33 U/L
Alanine Aminotransferase	212	7-55 U/L
Alkaline Phosphatase	825	44-147 U/L
Total Bilirubin	4.1	0.1-1.2 mg/dL
Direct Bilirubin	2.1	<0.3 mg/dL

**Figure 1:**
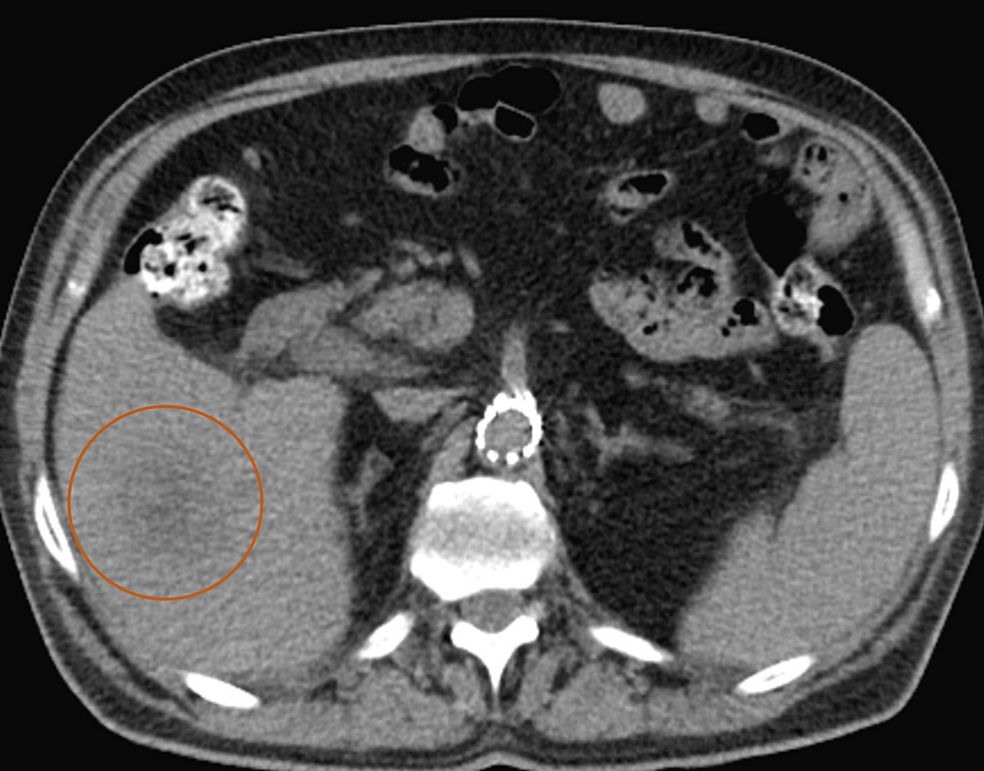
Computed tomography scan of the abdomen and pelvis without contrast demonstrating a new rounded ill-defined hypoattenuating lesion (circle) in the inferior aspect of the right liver lobe.

**Figure 2:**
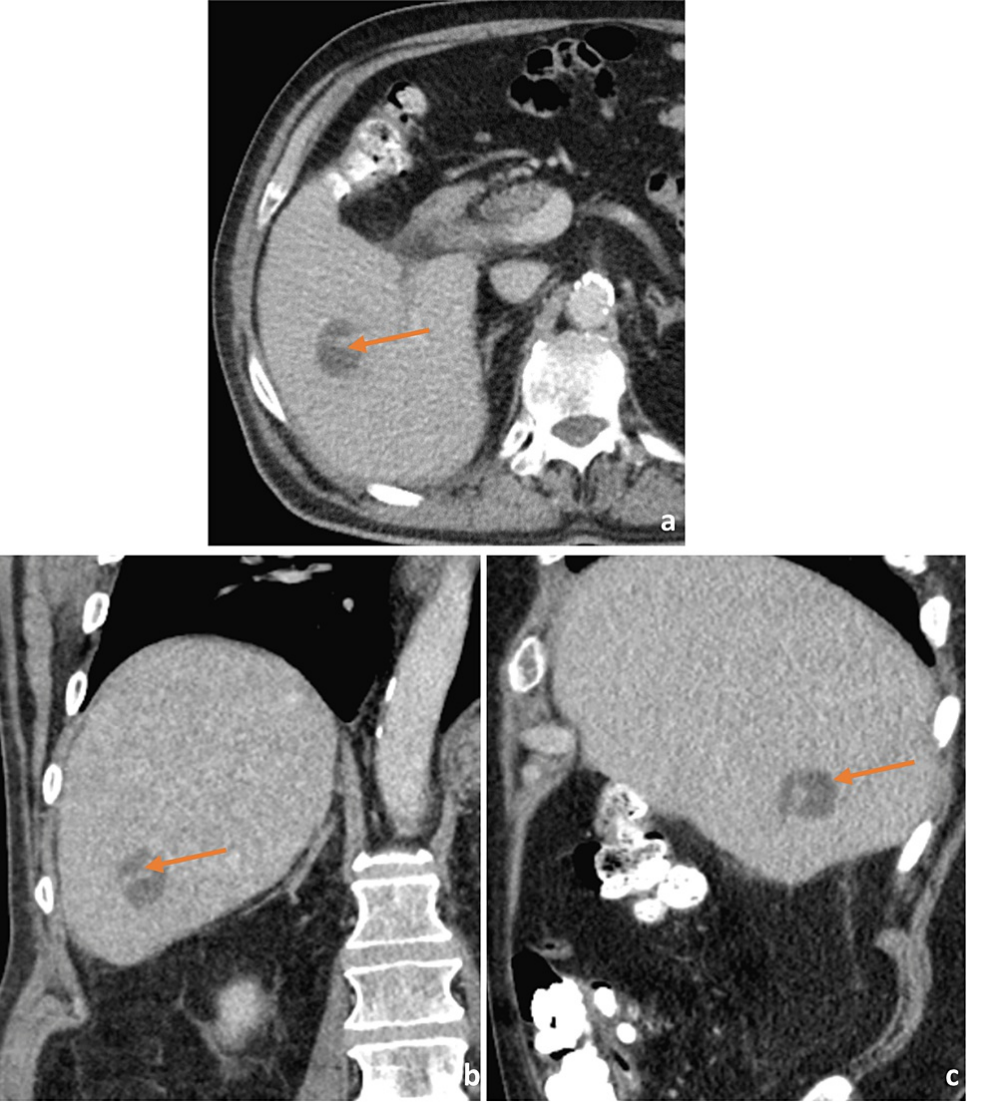
Triple phase computed tomography scan of the abdomen and pelvis in the transverse (a), coronal (b), and sagittal (c) planes demonstrating a 2.8 cm mass (arrows) in the right lobe of the liver with a small area of linear enhancement.

**Figure 3:**
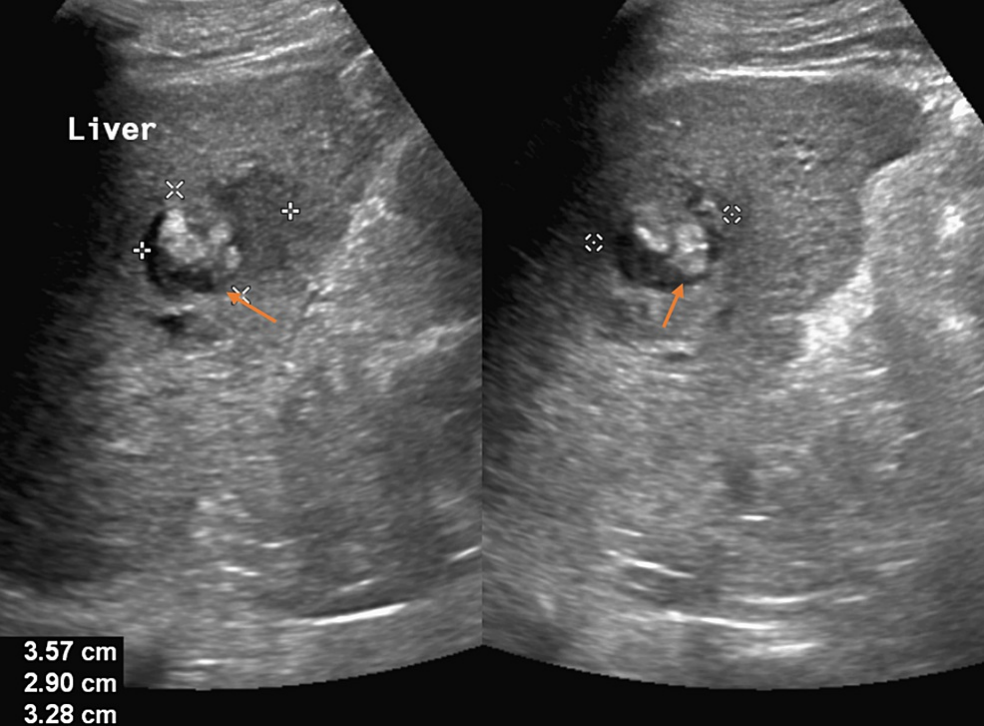
Abdominal ultrasound demonstrating an area of mixed echogenicity (arrows) with areas of increased and decreased sonographic texture measuring 3.6 x 2.9 x 3.3 cm in segment 8 of the liver.

Urinalysis was significant for dark yellow urine, protein of 30 (RR: negative), glucose of 200 (RR: negative), and bilirubin of +1 (RR: 0). Computed tomography (CT) scan of the abdomen and pelvis (A/P) without contrast demonstrated a rounded ill-defined low-attenuation lesion in the inferior aspect of the right lobe of the liver that is new since his most recent study (Figure 1). Triple-phase CT A/P was significant for a 2.8 cm mass in the right lobe of the liver with a small area of linear enhancement, possibly a complex cyst or abscess (Figures 2a-2c). Abdominal ultrasound (US) demonstrated an area of mixed echogenicity with areas of increased and decreased sonographic texture measuring 3.6 x 2.9 x 3.3 cm in segment 8 of the liver, without increased flow on Doppler (Figure 3).

Upon further evaluation of his medical record, the patient was noted to have had an *E. coli* abscess diagnosed 10 years prior, which was managed with antibiotics and percutaneous drainage by pigtail catheter. On imaging at that time, the abscess was situated in a similar position to that of the current abscess, within the right inferior part of the liver near segment 8. Abdominal imaging in the interim, after the management of the initial abscess and prior to this current episode, demonstrated no evidence of an existing intrahepatic fluid collection. As such, the initial abscess was deemed to have been treated with complete resolution.

The patient was managed with intravenous fluids to avoid post-contrast nephropathy. Infectious disease, gastroenterology, and interventional radiology (IR) were consulted. No antibiotics were started initially as the patient remained afebrile with no leukocytosis or any clinical signs of infection. LFTs continued to a downtrend during his hospital stay. The abscess was aspirated by IR, expressing purulent drainage. Abscess cultures subsequently grew gram-negative, oxidase-negative bacilli, representing *E. coli*. A liver biopsy was negative for malignancy. The patient was discharged with outpatient oral antibiotics and infectious disease follow-up.

## Discussion

PLAs can present asymptomatically or with non-specific symptoms; however, they have the potential for significant mortality and morbidity. Risk factors include diabetes mellitus and disorders of the hepatobiliary system. Complications include septic shock, abscess rupture resulting in peritonitis, pleural effusion, acute respiratory distress syndrome (ARDS), portal venous thrombosis, and resulting bacteremia with metastatic spread [6]. Increased mortality is associated with age 65 years old or greater, male sex at birth, presence of malignancy, presence of liver or biliary pathology, history of infection with multidrug-resistant organisms (MDROs), infection with *K. pneumoniae*, rupture, multiloculation, septic shock, organ failure and respiratory failure [7]. Despite a possible high mortality and multiple possible complications, PLA mortality has been steadily declining due to the implementation of abscess drainage as a standard of care in PLA management [4].

In terms of location, most PLAs are found within the right liver lobe, followed by the left lobe, and then least commonly within the caudate lobe [4]. The right lobe is dually supplied by the superior mesenteric vein and portal vein and is therefore more likely to be exposed to pathogenic bacteria. Since abscesses are most commonly located within the right liver lobe, they are viable candidates for percutaneous drainage.

PLA recurrence is overall rare, with an incidence of approximately 9%, with greater incidence in patients with biliary disease at over 20% [8]. Increased recurrence in PLA caused by biliary pathology is likely due to persistent re-colonization of the liver by pathogenic microorganisms found within the biliary tree. Recurrence is also more common in abscesses caused by extended-spectrum beta-lactamase (ESBL)-producing organisms, which are often brought about by prolonged hospitalizations, use of certain antibiotics (including fluoroquinolones and 3rd generation cephalosporins), and comorbid diabetes mellitus [9,10]. Recurrence is more common in PLAs caused by *K. pneumoniae* among diabetics [8]. In patients with biliary pathology, recurrence rates are not affected by the initial causative organism [8].

Upon diagnosis of a PLA, if no clear source can be delineated and if a patient has few risk factors, one must rule out malignancy as a potential cause [11]. Colorectal cancer, hepatocellular carcinoma, and biliary tract cancers have all been linked to the development of PLAs [11].

Here we describe a case of a patient with a history of diabetes and significant cardiovascular disease who presented following an episode of fever, dark urine, and jaundice, which resolved within 24 hours following admission, and who was found to have a PLA. The patient was otherwise non-toxic without complaints. This case was unique due to multiple factors. The patient was non-toxic and hemodynamically stable throughout his course, demonstrating an unusually mild case of PLA. Furthermore, he had a PLA 10 years prior, managed with antibiotics and drainage, found within the same location as the current abscess. This was remarkable in that abscess recurrence is rare in itself, and the identical distribution of both abscesses makes it rarer still. This was even more remarkable due to the long timeframe between both diagnoses. Documented cases of PLA recurrence show that abscesses most commonly recur within one year following the initial episode [12]. No significant studies demonstrate PLA recurrence multiple years after the resolution of the first abscess. Furthermore, this patient had no significant history of biliary disease that would predispose him to abscess development or re-development.

## Conclusions

PLAs can present asymptomatically, however, can carry devastating consequences if not managed promptly with antibiotics and drainage if needed. One must be cognizant of the comorbidities associated with liver abscesses, as well as risk factors for recurrence. Patients with no identifiable risk factors must be screened for a variety of malignancies associated with this disease. Here, we present a case of asymptomatic, recurrent PLA arising 10 years after the first incidence. Aspiration of the PLA revealed an identical causative organism to that of the first PLA. The patient was managed with antibiotics and discharged with outpatient follow-up.
